# Efficacy of Transarterial Chemoembolisation with or without Antiviral Therapy for Patients with Hepatocellular Carcinoma after Radical Hepatectomy

**DOI:** 10.1155/2018/6414759

**Published:** 2018-04-01

**Authors:** Yin Zhu, Pei-Jing Cui, Jing Yao, Zheng-Yun Zhang, Jun Yang

**Affiliations:** ^1^Department of Surgery, Affiliated Sixth People's Hospital, Shanghai Jiao Tong University, Shanghai 200233, China; ^2^Department of Geriatrics, Ruijin Hospital, School of Medicine, Shanghai Jiao Tong University, Shanghai 200025, China

## Abstract

**Objective:**

This study aimed at assessing the effects of transcatheter arterial chemoembolisation (TACE) and antiviral therapy on improving the prognosis of patients with hepatocellular carcinoma (HCC) after radical hepatectomy.

**Methods:**

This study reviewed the data of 120 patients with HCC who received either radical hepatectomy alone (control group), radical hepatectomy with postoperative TACE (TACE group) or radical hepatectomy with combined postoperative TACE, and antiviral therapy (combined group) from January 2000 to May 2015. To reduce the impact of the possible biases on the conclusion of this study to the minimum, the cases with similar demographic and clinicopathological characteristics were collected and 40 cases were assigned into each group. Recurrence, disease-free survival (DFS), and overall survival (OS) rates were compared.

**Results:**

Median follow-up period was 54.26 ± 22.65 months with a range of 17–110 months. Recurrence after radical surgery was observed for 39 (97.5%) patients in the TACE group, 32 (80%) in the combined group, and 40 (100%) in the control group with median recurrence duration of 33, 43, and 16.5 months, respectively. Postoperative TACE with or without antiviral therapy significantly prolonged the DFS rate compared with radical hepatectomy alone (*P* = 0.000). TACE combined with antiviral therapy significantly extended the DFS rate compared with TACE alone (*P* = 0.008). Postoperative TACE with or without antiviral therapy also significantly prolonged the OS rate compared with radical hepatectomy alone (*P* = 0.000). In addition, antiviral therapy combined with TACE significantly extended the 5-year OS rate of patients compared with individual TACE and radical hepatectomy (67.5% versus 55% and 2.5%; *P* = 0.032).

**Conclusion:**

TACE is an appropriate therapy for HCC patients after radical hepatectomy. When combined with antiviral therapy, this treatment may further prolong the recurrence time and thus lead to high DFS and OS rates.

## 1. Introduction

Hepatocellular carcinoma (HCC) is one of the most common malignancies worldwide. This disease accounts for 5.6% of all human cancers, and its incidence is gradually increasing [1]. In China, HCC mortality ranked second to stomach cancer. The main therapies for HCC are surgical interventions (tumour resection and liver transplantation), percutaneous interventions (ethanol injection and radiofrequency thermal ablation), transarterial interventions (embolisation, chemoperfusion, or chemoembolisation), and therapeutic drugs, including gene and immune therapy [2]. Among these treatments, surgical resection is the first-line therapeutic option for patients with HCC. Unfortunately, the long-term survival, even after curative liver resection, is unsatisfactory because of the high incidence of postoperative, specifically intrahepatic, tumour recurrence [3]. The 3- and 5-year recurrence rates are 62.3%–93.2% and 79.0%, respectively [4]. Several approaches were developed to decrease or delay the incidence of recurrence and the effects of HCC. As first reported by Goldstein, transcatheter arterial chemoembolisation (TACE) directly kills the tumour cells and blocks the blood supply to the tumour [5]. This treatment is now widely used in Chinese clinics to prevent the recurrence of HCC after surgery. However, its effects are still controversial and difficult to determine. Meta-analysis suggested that postoperative adjuvant TACE seems promising for participants with HCC with risk factors (multiple nodules of >5 cm or vascular invasion) [6]; however, other studies indicated that TACE can damage the remnant liver, harm the liver function, and interfere with immune function, which may increase the risk of reactivating hepatitis B virus (HBV) replication; all these effects can worsen the prognosis [7–9]. A study conducted in South Korea showed that TACE reactivates HBV replication in HCC patients that are HBsAg-positive. Higher HBV viral load may trigger HCC recurrence after liver resection [8]. Hung et al. [10] also found that a HBV viral load greater than 2000 IU/ml is associated with an odds ratio as high as 22.3 for HCC recurrence after liver resection. Antiviral therapy using nucleoside analogues is recently found to effectively supress HBV replication and reduce high viral load [11]. A small nonrandomised study from Japan found that lamivudine therapy is beneficial to patients that were initially treated with partial hepatectomy or radiofrequency ablation for HBV-related HCC [12]. Other studies showed that the combination of postoperative TACE and antiviral therapy may be more effective than TACE alone in improving the prognosis and reducing the recurrence in patients with HBV-related HCC after hepatectomy [13, 14].

Despite the increasing use of postoperative TACE and antiviral therapy for patients with HCC, a universally accepted guideline for the application in patients treated with radical hepatectomy is not yet established. Therefore, a retrospective review of the medical records of patients who received either radical hepatectomy alone, radical hepatectomy with postoperative TACE or radical hepatectomy with combined postoperative TACE, and antiviral therapy was conducted. The effects of TACE and antiviral therapy on improving the prognosis of patients with HCC after radical hepatectomy were assessed by comparing the recurrence, disease-free survival (DFS), and overall survival (OS) rates.

## 2. Methods

### 2.1. Patients

A total of 120 patients with chronic HBV infection were diagnosed with initial HCC in our department between January 2000 and May 2015. A retrospective analysis was conducted using the medical records of patients who satisfied the following inclusion criteria: (1) radical hepatectomy with histopathologically confirmed safety surgical margin of ≥2 cm; (2) histopathological examination confirming no major vascular or bile duct invasion or tumour thrombus in portal vein; (3) preoperative imaging diagnosis and surgical finding verifying no distant extrahepatic metastases; (4) chronic hepatitis B infection with HBV DNA level > 10^3^ copies/ml and without other viral hepatitis infections; (5) no evidence indicative of cancerisation from drug, alcoholic, fatty liver, or autoimmune hepatitis; and (6) without any other organ dysfunction or failure. Those patients who underwent preoperative treatments, such as TACE, antiviral therapy, or systemic chemotherapy, were excluded. Informed consent from all patients was obtained before performing any treatment.

### 2.2. Treatment

Hepatectomy was conducted by only one surgical team. Each patient received radical hepatectomy according to the anatomical principles of resection whenever possible. Intraoperative ultrasonography was used to identify any occult tumours that were not detected preoperatively and to confirm the relationship between the tumour and vasculobiliary structures. Parenchymal dissection was performed using ultrasonic dissectors.

### 2.3. Adjuvant TACE

TACE was conducted 2 months after radical hepatectomy by using the techniques described previously by Li et al. [15]. The therapeutic regimen included 5-fluorouracil (5-FU) 500 mg/m^2^, cisplatin (DDP) 40 mg/m^2^, and epirubicin (EPI) 40 mg/m^2^ with lipiodol 3 ml/m^2^ through chemoembolisation following hepatic arterial angiography. The puncture sites were compressed tightly, and the side limbs were restricted within a range of motion for at least 12 h. Supine position was assumed for 24 h afterwards to prevent bleeding or hematoma. The treatment was regularly repeated after 2 months depending on the patient's response to the first one.

### 2.4. Antiviral Therapy

This group includes patients who fulfilled the inclusion criteria and the following had ALT level two times higher than the upper limits and with or without HBV DNA greater than 10^4^ copies/ml, or with HBV DNA greater than 10^4^ copies/ml. These patients received lamivudine at a dose of 100 mg per day or entecavir 0.5–1.0 mg per day (if resistant to lamivudine) starting from 2 weeks after radical hepatectomy.

### 2.5. Follow-Up

The first endpoint was DFS, starting from the surgery date until the recurrence of HCC. The second one is OS, which is defined as the date of patient's death or last census date (1 May 2015). Patients were followed up to monitor HCC recurrence. Blood tests, including liver functions (ALT, AST, ALP, TB, and TSP), *α*-fetoprotein (AFP) level, and HBV DNA level, and ultrasonography were performed 1 month after surgery and then every 2 months afterwards for the first year. Intervals would later increase depending on the patient's situation. Further imaging studies such as CT or MRI were conducted if evidence of recurrence was observed. The diagnosis of recurrence or metastasis was confirmed under pathological examinations if necessary.

### 2.6. Statistical Analysis

SPSS 23.0 (SPSS Inc., Chicago, IL, USA) was used for statistical analysis. Demographic and clinical data were described using medians or frequencies. One-way ANOVA or chi-square test was used for the comparison of different groups. Fisher's exact test would be applied when homogeneity of variance assumption was satisfied. If the assumption failed, Brown-forsythe would be used. If *P* < 0.05 when comparing the demographic and clinical data, multiple comparisons like LSD test were used to distinguish the different one among the three groups. Regression analysis was used to determine the influencing factors. Survival curves were estimated using the Kaplan–Meier method and were compared through log-rank test. All statistical tests reached statistical significance at *P* < 0.05.

## 3. Results

### 3.1. Basic Demographic and Clinicopathological Characteristics of Patients

To reduce the impact of the possible biases on the conclusion of this study to the minimum, the cases with similar demographic and clinicopathological characteristics were collected. The records of 120 patients with HBV-related HCC treated in our department were retrospectively analysed. 40 cases were assigned into each group. Among them, 40 patients received radical hepatectomy alone (control group), 40 received radical hepatectomy and postoperative TACE (TACE group), and the remaining 40 received combination therapy of postoperative TACE and antiviral therapy after radical hepatectomy (combined group). The basic demographic and clinicopathological characteristics of patients before and after operation are shown in Tables 1 and 2. Among the three groups, no statistical significance (*P* > 0.05) was found for postoperative tumour characteristics and preoperative characteristics, which include the following: gender; age; counts of WBC, RBC, Hb, and PLT; and levels of ALT, AST, ALP, tumour markers (AFP, CA199, and CEA), and hepatitis B virus markers. Among the demographic characteristics, the patient number of ascites in the control group was different with that in the TACE group and combined group (*P* = 0.001, *P* = 0.000, resp.), and the total serum protein was different between the TACE group and combined group (*P* = 0.029), but the liver functions assessed by Child–Pugh class (consisting of ascites, hepatic encephalopathy, INR, total bilirubin, and total serum protein) showed no statistical significance (*P* > 0.05) (Supplementary Table 1). Median follow-up period was 54.26 ± 22.65 months with a range of 17–110 months.

### 3.2. Recurrence Rate of Patients in Different Groups

Recurrence after radical surgery was observed for 39 (97.5%) patients in the TACE group, 32 (80%) in the combined group, and 40 (100%) in the control group with median time of 33, 43, and 16.5 months, respectively. Most recurrence cases were intrahepatic, such as in 27 (67.5%) patients in the TACE group, 27 (67.5%) in the combined group, and 23 (57.5%) in the control group. Extrahepatic recurrence, mostly lung metastasis, was confirmed in 6 (15.0%) patients in the TACE group, 1 (2.5%) in the combined group, and 12 (30.0%) in the control group.

### 3.3. DFS of Patients in Different Groups

**Figure 1:**
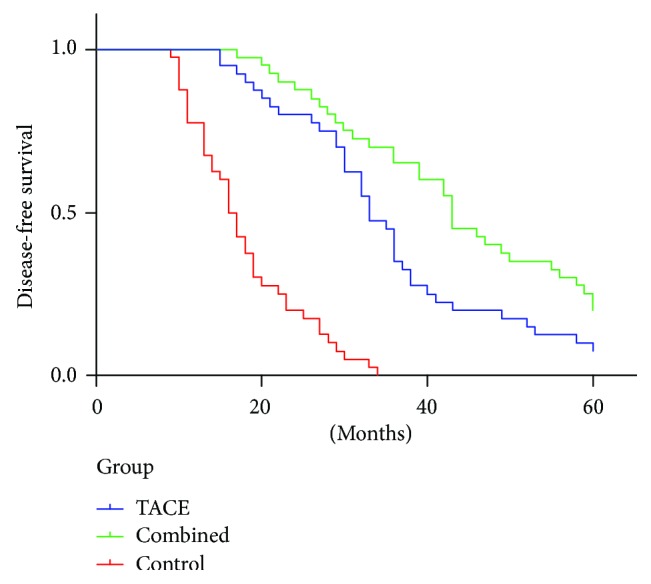
DFS of patients in different groups.

Figure 1 shows the DFS rates of different groups and reveals that postoperative TACE with or without antiviral therapy significantly prolonged the DFS rate compared with radical hepatectomy alone (*P* = 0.000). Compared with the above two treatments, TACE combined with antiviral therapy significantly extended the DFS of patients compared with TACE alone (*P* = 0.008). Therefore, postoperative TACE may be beneficial for the DFS of patients, and its combination with antiviral therapy could further increase this effect.

### 3.4. OS of Patients in Different Groups

**Figure 2:**
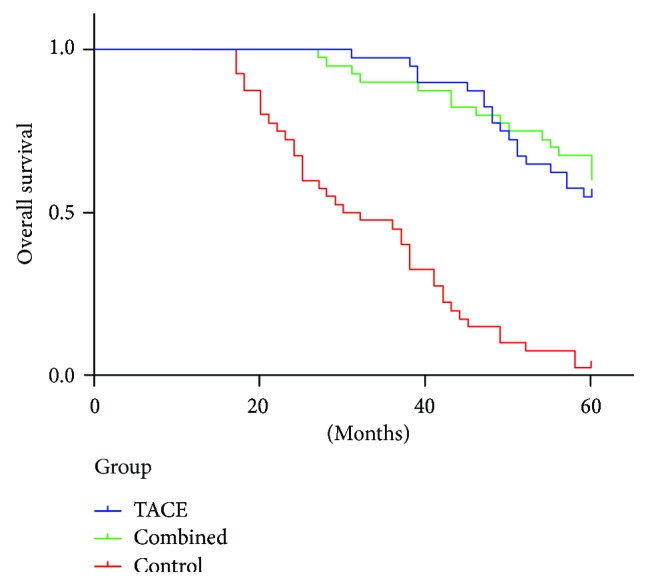
OS of patients in different groups.

Death due to HCC progression or other tumour complications occurred in 27 (67.5%) patients in the TACE group, 13 (32.5%) in the combined group, and all 40 (100%) patients in the control group with median OS rates of 64.50, 63.50, and 31 months, respectively. The OS rates of the three groups are presented in Figure 2. The combined group had significantly prolonged OS rate compared with the control group (*P* = 0.000). In addition, the 5-year OS rate of the combined group was significantly extended than that of the TACE and control groups (67.5% versus 55% and 2.5%; *P* = 0.032). Therefore, postoperative TACE and antiviral therapy may significantly prolong the OS rate of patients.

## 4. Discussion

HCC is the fifth most common malignancy in the world and causes approximately half a million deaths annually [16]. Surgical resection is the main treatment option for HCC. However, the high postoperative recurrence is the main obstacle for long-term survival. Various adjuvant treatments were developed with the hope of reducing recurrence rate and improving the OS of patients with HCC.

TACE has definite therapeutic effects for patients with inoperable HCC [17]. Compared with systemically administered drugs, TACE increases the concentration of local chemotherapy drug, resulting in good antitumour response and low incidence of systemic toxicity-related side effects [18]. However, the efficacy of postoperative TACE for patients with HCC treated with curative hepatectomy remains controversial. Some studies showed unsatisfactory results of this adjuvant treatment [7, 8]. A systematic review published in 2009 stated that no evidence suggests the efficacy of neoadjuvant/adjuvant protocols for the surgical resection of HCC [19]. The benefits of this treatment for postoperative patients require further studies. In addition, TACE might reactivate HBV replication after hepatectomy hypothetically due to its immunosuppression and cytotoxicity effects [20]. A related study also showed that the recurrence or metastasis rate dose dependently increases with the level of baseline HBV DNA; the range is from 22% for HBV DNA level of less than 3 log_10_ copies/ml to 80% for HBV DNA level of 5 log_10_ copies/ml or higher [21]. Therefore, effectively controlling HBV replication using antiviral therapy may lower the risk of recurrence after liver resection for patients with HCC [22]. The recently developed oral anti-HBV nucleoside analogs show promise with their ability to naturally suppress hepatitis. Antiviral therapy with nucleoside analogs effectively reduces HBV-induced liver damage, improves the liver function, promotes hepatocyte regeneration, and increases the volume of residual liver after hepatectomy [12, 23, 24]. However, limited credible evidence was found concerning the efficacy of combined postoperative TACE and antiviral therapy on HCC prognosis after radical tumour resection. Herein, we evaluated our medical data to explore the effect of postoperative TACE and antiviral therapy on the prognosis of HCC patients after radical hepatectomy.

Some authors suggested that adjuvant TACE is effective only for patients at high risk of recurrence due to multiple nodules and with >5 cm tumour size and vascular invasion and/or narrow resection margin [25–27]. In our current study, few patients have satellite nodules (4/40, 7/40, and 4/40), the maximum tumour size was >5 cm (4.31 ± 0.48 cm, 4.01 ± 0.67 cm, and 3.84 ± 0.68 cm), the resection margin exceeded 2.5 cm (2.60 ± 0.81 cm, 2.85 ± 1.05 cm, and 2.68 ± 1.02 cm), and no patient had vascular invasion. To the best of our knowledge, a consensus is not yet established with regard to several aspects of postoperative TACE [28]. Most HCC recurrent tumours appeared 2 years after surgery; hence, many scholars believe that adjuvant TACE after surgery should be conducted as soon as possible. Their recommended schedule is 1 month postoperation. In our study, we performed TACE 2 months after the surgery when the patients had recovered from the operation. In addition, the frequency of postoperative TACE administration remains controversial. Some scholars believe that if patients with a liver function can tolerate it, then TACE should be repeated throughout the recurrence peak period. However, other scholars believe that repeatedly performing TACE may increase hepatic dysfunction and a single TACE treatment is superior to the repeated therapy [29]. In our current study, we repeated TACE 2 months after surgery depending on the patient's response to the first one. Finally, no consensus was established for the suitable chemotherapeutic agents, dosage and rate of injection [28]. Based on our experience, we selected 5-FU, DDP, and EPI as the treatment protocol. We also mixed the chemotherapeutic drugs with lipiodol to help increase their viscosity and X-ray visibility, thereby prolonging the chemotherapy–tumour interaction.

Our study showed that patients treated with TACE had a low recurrence rate. Recurrences occur because of preexisting microscopic tumour foci that are undetected due to imaging modalities or disseminated malignant cells during surgical manipulation. Thus, postoperative adjuvant TACE aims at eliminating the shed tumour cells that are potentially released through surgical manipulation and to destroy small intrahepatic metastases that may not be preoperatively detected [6]. Our results were consistent with the previous studies stating that TACE can effectively reduce the postoperative recurrence rate. In addition, most of the recurrence cases in our study were localised intrahepatically but not extrahepatically after the postoperative TACE. These cases have high probability to be considered eligible for reoperation. Considering that high HBV load and mutants promote HCC metastasis and growth [10] whereas hepatitis B virus X protein promotes the invasive ability and metastatic potential of HCCs, the combined treatment of TACE and antiviral therapy can further reduce the possibility of recurrence via inhibiting these viral factors.

In addition to reducing the risk of recurrence, postoperative TACE with or without antiviral treatment prolonged the DFS and OS rates compared with the radical hepatectomy alone (*P* = 0.000). TACE with antiviral therapy significantly improved the DFS and OS rates relative to postoperative TACE alone. We therefore suggest that the combination of postoperative TACE and antiviral therapy may be more effective than TACE alone in improving HCC prognosis. This result is consistent with a previous study from China, which reported significantly high OS and DFS rates for patients treated with combined TACE and lamivudine [14]. However, another study showed that lamivudine cannot improve the DFS rate [24]. Theoretically, due to the immunosuppression and cytotoxicity of regional chemotherapy, HBV reactivation and replication may happen during the perioperative period, especially for patients who do not receive antiviral therapy. Administering lamivudine to HCC patients after radical hepatectomy may reduce the levels of HBV DNA in the circulation and the risk of liver failure, significantly improve the liver function, and increase the possibility that radical surgery can be repeated in the event of recurrence [30]. According to the literature review of Zhong et al. [31], antivirus therapy with nucleoside analogs should be recommended for the following: (i) patients who are in the decompensation stage of cirrhosis or those who have ALT levels that are twice higher than the upper normal limit, (ii) patients with compensated cirrhosis and serum concentrations of HBV DNA ≥ 10^4^ copies/ml (if HbeAg positive) or ≥10^3^ copies/mL (if HbeAg negative) regardless of the ALT level, and (iii) patients whose liver biochemistry findings are within the reference ranges when the serum concentrations of HBV DNA is >10^5^ copies/ml. In our study, we included patients with the above-mentioned criteria. Nucleoside analogs naturally prevent the deterioration of liver function and thus enhance the tolerance to subsequent therapy such as TACE. This phenomenon may be the reason why patients with combined therapy had good DFS rate. DFS is proposed as an alternative for OS. TACE combined with lamivudine prevents the recurrence of disease and simultaneously improves the DFS and OS rates.

Our findings suggest that TACE is an appropriate therapy for HCC patients after radical hepatectomy. When combined with antiviral therapy, this treatment may further prolong the time of recurrence, leading to good DFS and OS rates. However, due to the nonrandomisation and small sample size of our study, further randomised studies with larger numbers of patients and longer follow-up periods are necessary to clarify whether TACE combined with antiviral therapy can improve the prognosis of patients with HCC after radical hepatectomy.

## Figures and Tables

**Table 1:** Preoperative demographic characteristics of patients in the three groups.

Preoperative patient demographic		Control group	TACE group	Combined group	*P* value
Gender	Male	31 (77.5%)	26 (65%)	32 (80.0%)	0.263
	Female	9 (22.5%)	14 (35%)	8 (20.0%)	
Age (y)		52.25 ± 8.42	51.58 ± 8.26	49.78 ± 9.51	0.427
Ascites	Medically controlled	19 (47.5%)	5 (12.5%)	0 (0.0%)	0.000
	No	21 (52.5%)	35 (87.5%)	40 (100.0%)	
Hepatic encephalopathy	Yes	0 (0.0%)	0 (0.0%)	0 (0.0%)	1.000
	No	40 (100.0%)	40 (100.0%)	40 (100.0%)	
INR		1.06 ± 0.81	1.05 ± 0.75	1.06 ± 0.78	0.947
Total bilirubin (*μ*mol/L)		18.88 ± 4.29	19.53 ± 3.74	18.96 ± 4.14	0.739
Total serum protein (g/L)		35.93 ± 3.72	34.30 ± 1.96	35.80 ± 2.97	0.028
Child–Pugh class	A	33 (82.5%)	35 (87.5%)	36 (90.0%)	0.606
	B	7 (17.5%)	5 (12.5%)	4 (10.0%)	
ALT (U/L)		44.68 ± 9.96	50.00 ± 9.83	47.95 ± 12.48	0.090
ALP (U/L)		151.98 ± 70.39	149.10 ± 91.02	127.40 ± 70.52	0.308
AST (U/L)		40.70 ± 8.41	44.58 ± 10.11	43.08 ± 11.07	0.217
Cr (*μ*mol/L)		62.48 ± 13.06	60.93 ± 11.87	62.38 ± 12.32	0.823
WBC (×10^9^/L)		4.69 ± 0.57	4.88 ± 0.80	4.93 ± 7.95	0.304
RBC (×10^12^/L)		3.47 ± 0.36	3.32 ± 0.27	3.34 ± 0.33	0.100
PLT (×10^9^/L)		88.58 ± 19.61	90.48 ± 18.63	87.73 ± 23.37	0.830
Hb (g/L)		118.30 ± 18.89	113.48 ± 14.56	116.05 ± 14.16	0.406
AFP (ng/mL)		474.10 ± 185.44	949.80 ± 1160.62	710.76 ± 906.18	0.052
CEA (ng/mL)		2.15 ± 0.48	2.09 ± 0.52	2.24 ± 0.77	0.534
CA199 (ng/mL)		22.35 ± 4.43	20.80 ± 4.38	19.96 ± 4.66	0.058
HbsAg	Positive	40 (100.0%)	40 (100.0%)	40 (100.0%)	1.000
	Negative	0 (0.0%)	0 (0.0%)	0 (0.0%)	
HbsAb	Positive	7 (17.5%)	17 (42.5%)	16 (40.0%)	0.034
	Negative	33 (82.5%)	23 (57.5%)	24 (60.0%)	
HbcAb	Positive	40 (100.0%)	40 (100.0%)	40 (100.0%)	1.000
	Negative	0 (0.0%)	0 (0.0%)	0 (0.0%)	
HbeAg	Positive	40 (100.0%)	40 (100.0%)	40 (100.0%)	1.000
	Negative	0 (0.0%)	0 (0.0%)	0 (0.0%)	
HbeAb	Positive	40 (100.0%)	0 (0.0%)	0 (0.0%)	1.000
	Negative	0 (0.0%)	40 (100.0%)	40 (100.0%)	
HBA DNA level		2.93 × 10^5^	1.16 × 10^6^	5.66 × 10^5^	0.501

**Table 2:** Postoperative demographic characteristics of patients in the three groups.

Postoperative patient demographic		Control group	TACE group	Combined group	*P* value
Edmondson grade	I	1 (2.5%)	1 (2.5%)	1 (2.5%)	0.677
	II	16 (40.0%)	18 (45.0%)	20 (50.0%)	
	III	15 (37.5%)	14 (35%)	13 (32.5%)	
	IV	8 (20.0%)	7 (17.5%)	6 (15.0%)	

AJCC tumour stage	I	36 (90.0%)	34 (85.0%)	37 (92.5%)	0.549
	II	4 (10.0%)	6 (15.0%)	3 (7.5%)	

Tumour number	1	36 (90.0%)	35 (87.5%)	37 (92.5%)	0.759
	2	4 (10.0%)	5 (12.5%)	3 (7.5%)	

Maximum tumour size (cm)		4.31 ± 0.48	4.01 ± 0.67	3.84 ± 0.68	0.004

Fibrous capsule formation	Yes	4 (10.0%)	5 (12.5%)	6 (15.0%)	0.797
	No	36 (90.0%)	35 (87.5%)	34 (85.0%)	

Tumour invasion in capsule	Yes	0 (0.0%)	4 (10.0%)	3 (7.5%)	0.141
	No	40 (100.0%)	36 (90.0%)	37 (92.5%)	

Microvascular invasion	Yes	6 (15.0%)	8 (20.0%)	4 (10.0%)	0.459
	No	34 (85.0%)	32 (80.0%)	36 (90.0%)	

Portal vein invasion	Yes	0 (0.0%)	0 (0.0%)	0 (0.0%)	1.000
	No	40 (100.0%)	40 (100.0%)	40 (100.0%)	

Serosa invasion	Yes	0 (0.0%)	1 (2.5%)	0 (0.0%)	0.368
	No	40 (100.0%)	39 (97.5%)	40 (100.0%)	

Satellite nodule	Yes	4 (10.0%)	7 (17.5%)	4 (10.0%)	0.507
	No	36 (90.0%)	33 (82.5%)	36 (90.0%)	

Surgical margin invasion	Yes	0 (0.0%)	0 (0.0%)	0 (0.0%)	1.000
	No	40 (100.0%)	40 (100.0%)	40 (100.0%)	

Safety margin (cm)		2.60 ± 0.81	2.85 ± 1.05	2.68 ± 1.02	0.497

p53	Positive	16 (40.0%)	19 (47.5%)	20 (50.0%)	0.649
	Negative	24 (60.0%)	21 (52.5%)	20 (50.0%)	

## Supplementary Materials

Supplementary Materials Supplementary materials for supplementary Table 1: patients' demographic data were retrospectively collected from the database. Demographic data were described using medians or frequencies. One-way ANOVA or chi-square test was used for the comparison of different groups. All statistical tests reached statistical significance at *P* < 0.05.
