# Supplementary material for: Efficacy of Transarterial Chemoembolisation with or without Antiviral Therapy for Patients with Hepatocellular Carcinoma after Radical Hepatectomy
Source: Gastroenterol Res Pract. 2018 Apr 1;2018:6414759. doi: 10.1155/2018/6414759 (PMC5902052; doi:10.1155/2018/6414759)
Supplement: Supplementary Materials — for supplementary Table 1: patients' demographic data were retrospectively collected from the database. Demographic data were described using medians or frequencies. One-way ANOVA or chi-square test was used for the comparison of different groups. All statistical tests reached statistical significance at P < 0.05. [file 6414759.f1.doc]

Supplementary Table.1 Comparison of patients’ demographic characteristics of among the three groups

| Patient Demographic | Group | Group | P value |
| --- | --- | --- | --- |
| Age (y) | TACE | Combined | 0.359 |
|  |  | Control | 0.731 |
|  | Combined | Control | 0.208 |
| Gender | TACE | Combined | 0.323 |
|  |  | Control | 1.000 |
|  | Combined | Control | 0.210 |
| Ascites | TACE | Combined | 0.055 |
|  |  | Control | 0.001 |
|  | Combined | Control | 0.000 |
| Hepatic encephalopathy | TACE | Combined | 1.000 |
|  |  | Control | 1.000 |
|  | Combined | Control | 1.000 |
| INR | TACE | Combined | 0.775 |
|  |  | Control | 0.775 |
|  | Combined | Control | 1.000 |
| Total bilirubin (μmol/L) | TACE | Combined | 0.532 |
|  |  | Control | 0.478 |
|  | Combined | Control | 0.932 |
| Total serum protein (g/L) | TACE | Combined | 0.029 |
|  |  | Control | 0.052 |
|  | Combined | Control | 0.998 |
| Child–Pugh class | TACE | Combined | 1.000 |
|  |  | Control | 0.755 |
|  | Combined | Control | 0.518 |
| ALT (U/L) | TACE | Combined | 0.399 |
|  |  | Control | 0.030 |
|  | Combined | Control | 0.179 |
| ALP (U/L) | TACE | Combined | 0.215 |
|  |  | Control | 0.869 |
|  | Combined | Control | 0.161 |
| AST (U/L) | TACE | Combined | 0.500 |
|  |  | Control | 0.083 |
|  | Combined | Control | 0.287 |
| Cr (μmol/L) | TACE | Combined | 0.603 |
|  |  | Control | 0.578 |
|  | Combined | Control | 0.971 |
| WBC (×10^9^/L) | TACE | Combined | 0.760 |
|  |  | Control | 0.247 |
|  | Combined | Control | 0.144 |
| RBC (×10^12^/L) | TACE | Combined | 0.851 |
|  |  | Control | 0.052 |
|  | Combined | Control | 0.079 |
| PLT (×10^9^/L) | TACE | Combined | 0.552 |
|  |  | Control | 0.681 |
|  | Combined | Control | 0.854 |
| Hb (g/L) | TACE | Combined | 0.473 |
|  |  | Control | 0.180 |
|  | Combined | Control | 0.531 |
| AFP (ng/mL) | TACE | Combined | 0.215 |
|  |  | Control | 0.014 |
|  | Combined | Control | 0.219 |
| CEA (ng/mL) | TACE | Combined | 0.268 |
|  |  | Control | 0.670 |
|  | Combined | Control | 0.494 |
| CA199 (ng/mL) | TACE | Combined | 0.408 |
|  |  | Control | 0.125 |
|  | Combined | Control | 0.063 |
| HbsAg | TACE | Combined | 1.000 |
|  |  | Control | 1.000 |
|  | Combined | Control | 1.000 |
| HbsAb | TACE | Combined | 1.000 |
|  |  | Control | 0.027 |
|  | Combined | Control | 0.047 |
| HbcAb | TACE | Combined | 1.000 |
|  |  | Control | 1.000 |
|  | Combined | Control | 1.000 |
| HbeAg | TACE | Combined | 1.000 |
|  |  | Control | 1.000 |
|  | Combined | Control | 1.000 |
| HbeAb | TACE | Combined | 1.000 |
|  |  | Control | 1.000 |
|  | Combined | Control | 1.000 |
| HBA DNA level | TACE | Combined | 0.775 |
|  |  | Control | 0.398 |
|  | Combined | Control | 0.259 |
| Edmondson grade | TACE | Combined | 0.974 |
|  |  | Control | 0.974 |
|  | Combined | Control | 0.832 |
| AJCC tumour stage | TACE | Combined | 0.481 |
|  |  | Control | 0.737 |
|  | Combined | Control | 1.000 |
| Tumour number | TACE | Combined | 0.712 |
|  |  | Control | 0.723 |
|  | Combined | Control | 0.692 |
| Maximum tumour size (cm) | TACE | Combined | 0.222 |
|  |  | Control | 0.034 |
|  | Combined | Control | 0.001 |
| Fibrous capsule formation | TACE | Combined | 0.745 |
|  |  | Control | 0.723 |
|  | Combined | Control | 0.737 |
| Tumour invasion in capsule | TACE | Combined | 0.712 |
|  |  | Control | 0.055 |
|  | Combined | Control | 0.241 |
| Microvascular invasion | TACE | Combined | 0.348 |
|  |  | Control | 0.770 |
|  | Combined | Control | 0.737 |
| Portal vein invasion | TACE | Combined | 1.000 |
|  |  | Control | 1.000 |
|  | Combined | Control | 1.000 |
| Serosa invasion | TACE | Combined | 0.314 |
|  |  | Control | 0.314 |
|  | Combined | Control | 1.000 |
| Satellite nodule | TACE | Combined | 0.518 |
|  |  | Control | 0.518 |
|  | Combined | Control | 1.000 |
| Surgical margin invasion | TACE | Combined | 1.000 |
|  |  | Control | 1.000 |
|  | Combined | Control | 1.000 |
| Safety margin (cm) | TACE | Combined | 0.420 |
|  |  | Control | 0.250 |
|  | Combined | Control | 0.729 |
| p53 | TACE | Combined | 0.823 |
|  |  | Control | 0.499 |
|  | Combined | Control | 0.369 |
